# Determination of Isoflavones in *Radix puerariae* from Different Origins by Ultra-High Performance Liquid Chromatography Based on Optimal Pretreatment Method

**DOI:** 10.3390/foods12040794

**Published:** 2023-02-13

**Authors:** Yuan Gao, Mengjia Xu, Hao Wan, Chang Li, Yiqun Wan

**Affiliations:** 1 College of Chemistry and Chemical Engineering, Nanchang University, Nanchang 430047, China; 2 State Key Laboratory of Food Science and Technology, Nanchang University, Nanchang 330047, China

**Keywords:** *Radix puerariae*, isoflavones, dispersed solid phase purification, ultra-high performance liquid chromatography

## Abstract

A new method for simultaneous determination of puerarin, daidzin, daidzein and genistein in *Radix puerariae* by ultra-high performance liquid chromatography was established. The target analytes were extracted from *Radix puerariae* by 70% ethylene glycol with the assistance of ultrasonication, purified by the absorption of N-propyl ethylenediamine (PSA), and separated on a Supersil ODS column (4.6 mm × 250 mm × 2.5 μm). Gradient elution in 12 min was performed with the mobile phase 0.1% formic acid(A)–acetonitrile(B). The column temperature was 25 °C and the flow rate was 1 mL/min. The detection wavelength of the four target analytes was 250 nm. The limits of detection (LODs) of puerarin, daidzin, daidzein and genistein were 0.086 mg/L, 0.020 mg/L, 0.027 mg/L and 0.037 mg/L, respectively, and limits of quantitation (LOQs) were 0.29 mg/L, 0.065 mg/L, 0.090 mg/L and 0.12 mg/L, respectively. The recovery of the four substances ranged from 90.5% to 109.6%, and the relative standard deviation (*n* = 6) was less than 7.7%. With the established methods, puerarin, daidzin, daidzein and genistein in *Radix puerariae* from 11 origins were determined. The contents of the four compounds varied with the origin and variety. It provides basic data and technical means for quality control and regulation of *Radix puerariae*.

## 1. Introduction

*Radix puerariae* is the tuber root of the leguminous plant Puerariae, which was originally recorded in Shennong Bencao Jing (an ancient Chinese medical book) in ancient China [1]. It is a twining perennial woody herb found in China, Korea, Japan, India and the United States. It is also known as the “invasive species” by Americans, and the US government spends billions of dollars to eliminate it every year. *Radix puerariae* is mainly found in Yunnan, Anhui, Sichuan, Jiangxi, Guangxi, Guangdong, Hainan provinces and other places in China. As a recognized nutritive plant, *Radix puerariae* is used not only in food but also in medicine in China. As many as 90 prescriptions for *Radix puerariae* are listed in the 2020 edition of the Chinese Pharmacopoeia, and modern pharmaceutical companies have developed numerous products. In terms of food, *Radix puerariae* has been developed into some products such as noodles, freshly squeezed drinks and bread due to its high starch content and unique taste [2]. With the wide range of applications of *Radix puerariae*, its safety has been of great concern. Wang et al. reported that *Radix puerariae* extract may be hepatotoxic [3]. Studies have shown that puerarin in *Radix puerariae* can cause acute renal failure, hepatotoxicity and hemolytic reactions [3,4]. The developmental and reproductive toxicity of isoflavones in *Radix puerariae* has also been reported [5].

In addition, *Radix puerariae* contains isoflavones, triterpenes, coumarins, glycosides and other components [6]. Of these, isoflavones are the main active components, with relatively high levels of puerarin, daidzein, daidzein and genistein [7], which are commonly used as active components in the quality control of *Radix puerariae* [6,8,9]. The isoflavones in *Radix puerariae* are phenolic phytoestrogens, and due to their structural similarity to estradiol, they may interfere with the endocrine system, posing a health risk to humans [10,11]. As a result, the safety of *Radix puerariae* has received widespread attention.

At present, the contents of isoflavones in *Radix puerariae* are determined by high-performance liquid chromatography (HPLC) with a UV, fluorescence or mass spectrometric detector [12,13,14]. Due to the complexity of *Radix puerariae* matrix, there are still many interfering substances in the extract [15], which not only affect the detection of the target analytes but also shorten the service life of the chromatographic column [16,17]. Extracts of *Radix puerariae* can be purified by some adsorbents prior to injection for analysis. Purification adsorbents such as HC-C18 [18], silica gel [19], primary secondary amine (PSA) [20], kieselguhr [21] and ZIF-8 [22] have been widely applied to remove the interference in other studies. However, there are few reports on the purification of samples using those absorbents in the determination of isoflavones in *Radix puerariae*.

The aim of this research was to establish a new UHPLC method for the simultaneous analysis of puerarin, daidzin, daidzein and genistein in *Radix puerariae* and to attempt to optimize the sample pretreatment with five adsorbents. Furthermore, four isoflavones in *Radix puerariae* from different origins were determined with the developed method.

## 2. Materials and Methods

### 2.1. Chemicals and Reagents

Methanol, ethylene glycol and formic acid (analytically pure) were purchased from Xilong Science Co., Ltd. (Shantou, China). Acetonitrile (HPLC grade) was purchased from TEDIA Co., Ltd. (Fairfield, OH, USA). Ethanol (chromatographic pure) was bought from Xilong Science Co., Ltd. (Shantou, China). Puerarin, daidzin, daidzein and genistein (HPLC grade) were obtained from Shanghai Yuanye Biotechnology Co., Ltd. (Shanghai, China). Puerarin, daidzin, daidzein and genistein were prepared into 1000 mg/L standard stock solution with methanol. The stock solution was diluted to prepare a series of mixed standard working solutions as required.

### 2.2. Sample Pretreatment

The dried *Radix puerariae* sample was crushed and sieved through a fine screen. Approximately 0.5 g of sample powder was accurately weighed and placed in a 50 mL centrifuge tube, then 25 mL 70% ethylene glycol aqueous solution was added, and the sample was mixed by a vortexer (Ika, Staufen, Germany). After ultrasonic extraction at 25 °C for 40 min, the centrifugation was performed at 4000 r/min for 5 min in a centrifuge (3K15, Sigma Laboratory Centrifuges, Osterode, Germany). One milliliter of the extract was added to 4 mL of 70% ethylene glycol aqueous solution and 50 mg PSA adsorbent. After shaking for 30 min, the mixture was centrifuged at 4000 r/min for 5 min. One milliliter of the supernatant was filtered through 0.22 μm organic microporous membrane and stored at 4 °C until measurement.

### 2.3. UPLC Condition

Ultra-high performance liquid chromatography (UHPLC) analysis was performed using an Agilent 1290 ultra-performance liquid chromatograph (Agilent, Santa Clara, CA, USA) equipped with high-pressure binary pumps and a diode array detector (DAD). The chromatographic column was Supersil ODS (4.6 mm × 250 mm × 2.5 μm). The column temperature was 25 °C. The mobile phase was 0.1% formic acid (A) and acetonitrile (B). The gradient elution procedure is shown in Table 1. The flow rate was 1 mL/min. The detection wavelength was 250 nm and the injection volume was 10 μL.

### 2.4. Statistical Analysis

Microsoft Excel (v.2019, Microsoft corporation, Redmond, WA, USA) was used for the statistical processing of all data, SPSS 20.0 (IBM, Chicago, IL, USA) was used for the analysis of variance, and the LSD method was used to test the significance of difference between different means.

## 3. Results and Discussion

### 3.1. Optimization of Chromatographic Conditions

**Figure 1:**
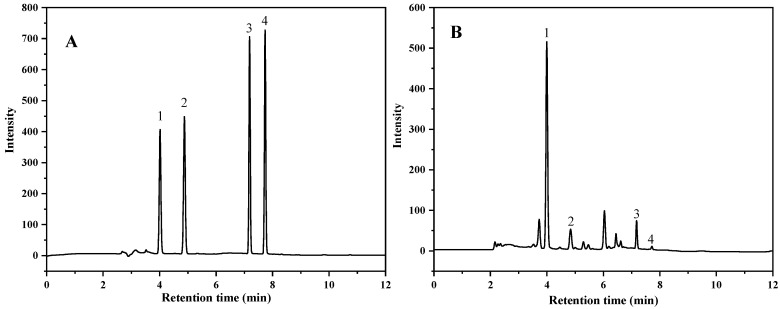
UHPLC chromatogram of the standards (**A**) (50 mg/L) and the sample (**B**) (1. Puerarin; 2. Daidzin; 3. Daidzein; 4. Genistein) (sample was purified with PSA).

In this study, three different mobile phase combinations (including 0.3% formic acid-methanol, 0.3% formic acid-acetonitrile and 0.1% formic acid-acetonitrile) and the gradient elution procedures were optimized. The results showed that puerarin, daidzin, daidzein and genistein could be separated by gradient elution using the combination of 0.1% formic acid (A) and acetonitrile (B) as the mobile phase in 12 min. The UHPLC chromatogram of four compounds is shown in Figure 1. It was previously reported that the separation of these four compounds by HPLC required at least 40 min [23] or even longer than 80 min [24]. The condition optimized in this study is advantageous in terms of gradient time.

### 3.2. Optimization of Extraction Conditions

**Figure 2:**
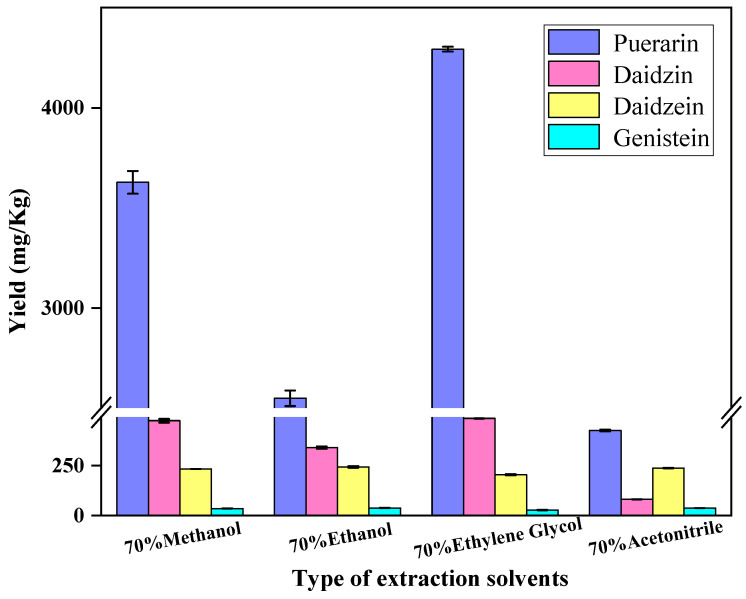
Effect of the extraction solvent on the yield of puerarin, daidzin, daidzein and genistein.

Methanol, ethanol and acetonitrile are commonly used to extract isoflavones [12,25,26,27]. Extraction using solvent alone is often inefficient. Ultrasound methods have been identified as the most cost-effective way to increase the yield of isoflavones with lower time and solvent costs at lower temperatures [28]. In this study, the extraction for testing samples was performed using four solvents with the assistance of ultrasonication, and the extraction yields were compared. Briefly, 0.5 g of *Radix puerariae* samples were weighed and 20 mL of 70% methanol, 70% ethanol, 70% ethylene glycol and 70% acetonitrile were selected as the extraction solvent; after being vortexed, the samples were extracted with the assistance of ultrasonication at 25 °C for 30 min, then the extracts were centrifuged at 4000 r/min for 5 min. The extraction yields are shown in Figure 2. The 70% ethylene glycol exhibited the most effectiveness in terms of extraction yield. Therefore, 70% ethylene glycol was selected as the extraction solvent in this study.

**Figure 3:**
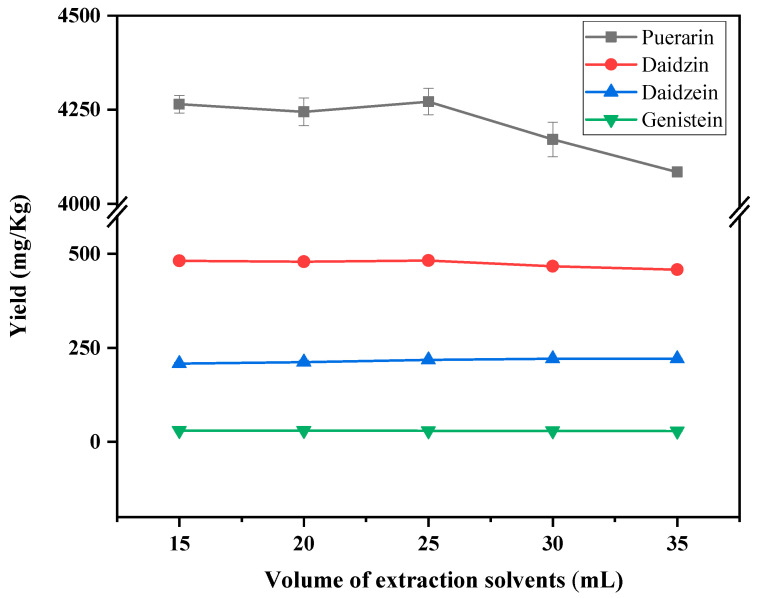
Effect of ethylene glycol dosage on the yield of puerarin, daidzin, daidzein and genistein.

The effect of the amount of ethylene glycol solution was investigated on the extraction yield of puerarin, daidzin, daidzein and genistein. The results are shown in Figure 3. The extraction effects of daidzein and genistein did not change much among different extraction solvent doses. Puerarin and daidzein demonstrated the best extraction yields when the extraction solvent dosage was 25 mL, so the extraction solvent dosage was chosen to be 25 mL.

**Figure 4:**
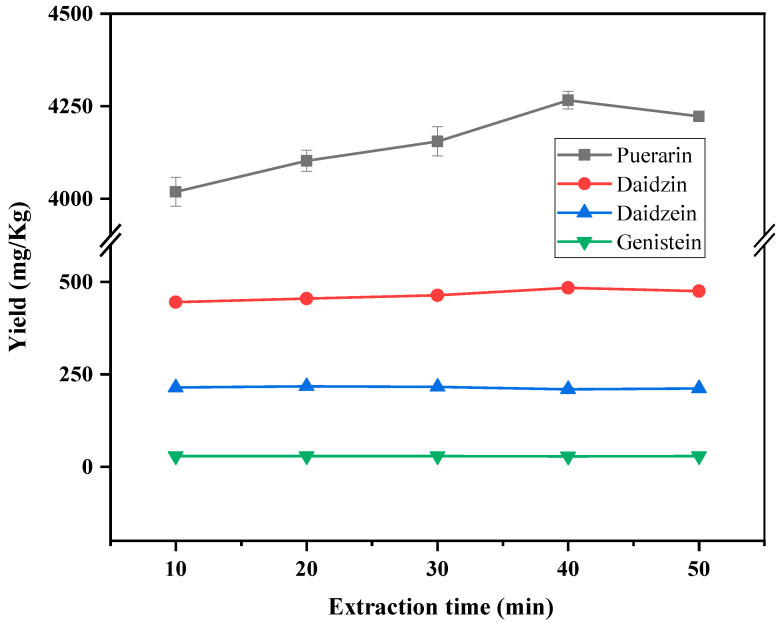
Effect of the extraction time on the yield of puerarin, daidzin, daidzein and genistein.

The effect of extraction time in the range of 10–50 min on the yield of target compounds was also investigated. The results are shown in Figure 4. Daidzein and genistein showed little change in yield within 10–50 min. The yields of puerarin and daidzein tended to be stable after 40 min of ultrasonic time. Therefore, the ultrasonic extraction time was selected to be 40 min.

### 3.3. Optimization of Purification Conditions

**Figure 5:**
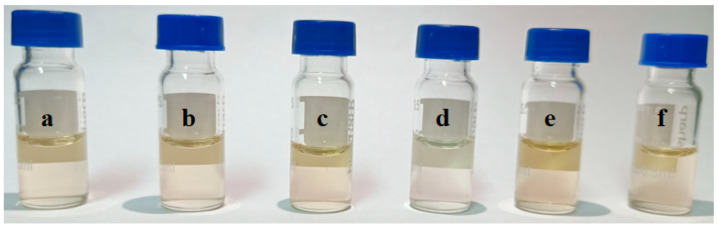
Purification effect of different adsorbents on the extract: (**a**) no material added; (**b**) HC-C18; (**c**) diatomite; (**d**) PSA; (**e**) ZIF-8; and (**f**) silica gel.

**Figure 6:**
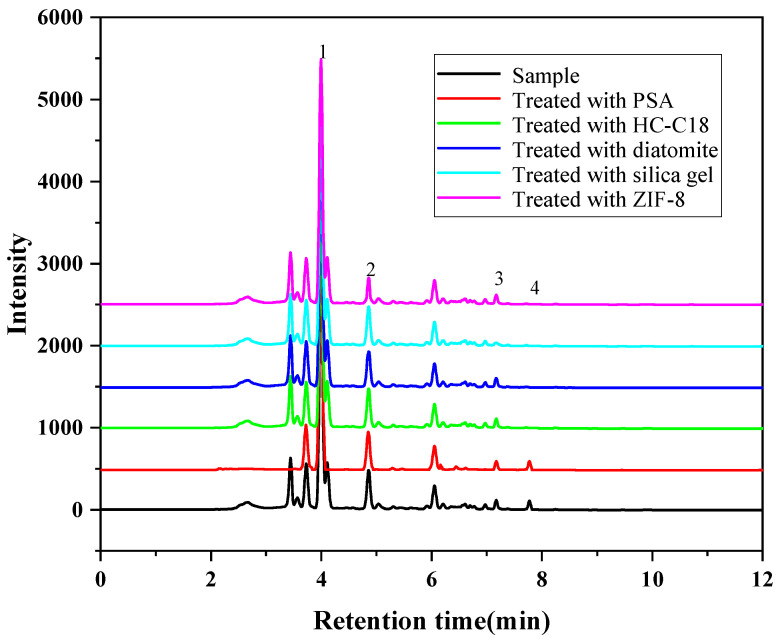
Purification effect of different adsorbents on the extract (1. Puerarin; 2. Daidzin; 3. Daidzein; 4. Genistein).

Considering the complexity of the extract of the *Radix puerariae* sample, some matrix compounds may interfere with the detection of the target analytes and shorten the service life of the column. For instance, pigments in the extract often interfere with the detection of the target analytes [29,30]. Therefore, several solid adsorbents were investigated in this experiment to remove the interfering substances in the extract. The sorbent type, dosage and adsorption time were optimized. Firstly, the purification effects of HC-C18, silica gel, PSA, diatomite and ZIF-8 on the extract were investigated. As shown in Figure 5, PSA has the best pigment removal effect compared with other adsorbents. Then the purification liquid was determined by high-performance liquid chromatography, and the results are shown in Figure 6, PSA has the best purification effect on the extract, which can remove the impurities and remain genistein compared with the other adsorbents. Furthermore, other adsorbents will have an adsorption effect on genistein. Therefore, PSA was chosen as purifying sorbent. The purification effect was investigated for PSA doses in the range of 10 to 300 mg. The results showed that the decolorization effect did not change significantly when the PSA dosage was greater than 50 mg. However, as the PSA dose was increased beyond 50 mg, the PSA showed an adsorption effect on the target analyte. Therefore, the PSA dosage was selected as 50 mg. In addition, the adsorption time in the range of 10–50 min was investigated. The optimal purification was obtained when the treatment was continued for 20 min. Therefore, the adsorption time was chosen to be 20 min in the present study.

### 3.4. Linearity, Limits of Detection and Quantification

A series of mixed standard operating solutions with different concentrations were prepared and measured under optimized experimental conditions. The peak area of each component, Y, was linearly fitted to the concentration of the analyte, X, to obtain the operating curve, and the results are presented in Table 2. The limits of detection (LODs) and limits of quantification (LOQs) values of the investigated analytes were assessed using signal-to-noise (S/N) ratios of 3 and 10, respectively; the results are shown in Table 2. The linear correlation coefficient (R^2^) of the analyte was better than 0.9981, indicating good linearity. Puerarin, daidzin, daidzein and genistein had LODs of 0.086 mg/L, 0.020 mg/L, 0.027 mg/L and 0.037 mg/L, respectively, and LOQs of 0.29 mg/L, 0.065 mg/L, 0.090 mg/L and 0.12 mg/L, respectively.

### 3.5. Recovery and Precision

**Figure 7:**
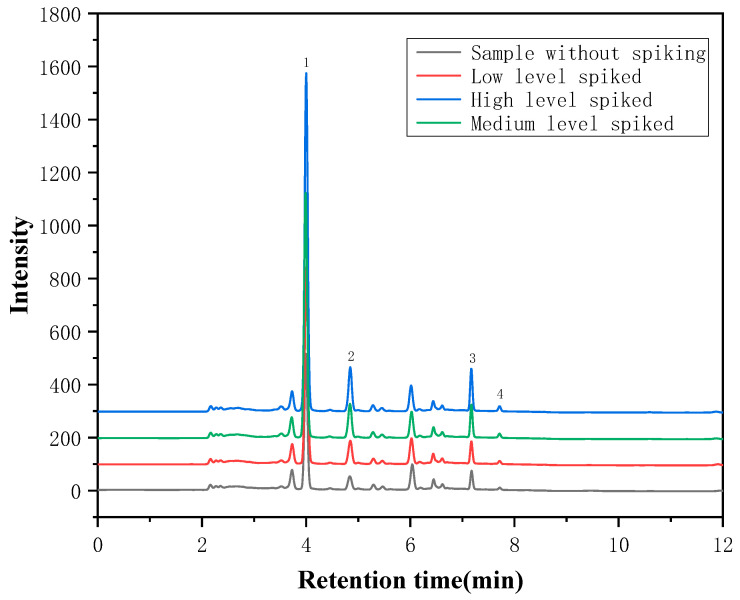
The chromatogram of the samples with and without spiked standards (1. Puerarin; 2. Daidzin; 3. Daidzein; 4. Genistein).

Three mixed standard solutions with different (Low, Medium and High) concentration levels were added to the samples, respectively, to carry out the spiked recovery experiment, and each level was measured in parallel six times. The results are shown in Table 3. The recoveries of the target analytes were 96.1–102.2% for puerarin, 97.6–109.6% for daidzin, 94.9–104.7% for daidzein, and 90.5–102.2% for genistein, respectively, and the relative standard deviation (RSD, *n* = 6) was less than 7.7%, indicating that this method has good precision, and can be used for the analysis of actual samples. Moreover, as shown in Figure 7, the measured concentration increases and the peak position remains the same when different standard concentrations are added. This indicates that the tested compounds are not interfered with by the matrix.

### 3.6. Determination of the Sample

Four target compounds in *Radix puerariae* samples from 11 different regions of China were determined by the established analytical method, and the results are shown in Table 4. The levels of four compounds in the *Radix puerariae* samples from different regions were varied. The highest level of puerarin was detected in the sample from Chengdu, Sichuan, which was 101.9 mg/g. The lowest level of puerarin was found in the sample from Bozhou, Anhui, at only 4.14 mg/g. The highest level of daidzein was found in the sample from Linyi, Shandong, as high as 1.36 mg/g. The content differences of the compounds are attributed to the variety of *Radix puerariae*. *Radix puerariae lobata* (Wild.) *Ohwi* (RPL) and *Radix puerariae thomsonii Benth* (Fabaceae family) (RPT) are two varieties of *Radix puerariae* [31]. The collected samples contain both varieties of RPL and RPT (Table 4). Notably, the levels of puerarin, daidzin and daidzein in RPL are much higher than those in RPT. In the previous study, puerarin, daidzin and daidzein were confirmed to be the markers to distinguish RPL and RPT [12]. RPT is rich in starch and is commonly used as food, and contains fewer isoflavones than RPL [15,31]. RPL is commonly used as a herb in China. The highest levels of puerarin and daidzin were detected in RPL samples collected from Chengdu, Sichuan province. Among the RPT samples, those from Chuxiong, Yunnan province, were found to have the highest levels of puerarin and daidzin. On the whole, the genistein content in RPL is higher than that in RPT, except in the sample from Shennongjia, Hubei (Table 4). 

## 4. Conclusions

In the present study, a UHPLC method with the optimal pretreatment of the sample was established for the simultaneous determination of puerarin, daidzin, daidzein and genistein in *Radix puerariae*. PSA is the most effective absorbent for sample purification. The method is simple, rapid, accurate and suitable for the determination of puerarin, daidzin, daidzein and genistein in *Radix puerariae* samples. With the established methods, puerarin, daidzin, daidzein and genistein in *Radix puerariae* from 11 origins in China were determined. Levels of four compounds in *Radix puerariae* vary with the origin. The levels of puerarin, daidzein and daidzin are much higher in RPL than in RPT. Puerarin, daidzein and daidzin may be the indicators of discrimination of RPL and RPT.

## Figures and Tables

**Table 1:** The gradient elution procedure.

Time (min)	A (%)	B (%)
0	80	20
3	70	30
6	20	80
10	20	80
10.1	80	20

**Table 2:** Linear equation, detection limit and quantification limit of puerarin, daidzin, daidzein and genistein.

Compounds	Linear Range (mg/L)	Calibration Curves	R^2^	LOD (mg/L)	LOQ (mg/L)
Puerarin	2–200	*y* = 25.6*x* − 38.6	0.9981	0.086	0.29
Daidzin	1–100	*y* = 26.7*x* − 5.6	0.9981	0.020	0.065
Daidzein	1–100	*y* = 57.0*x* − 22.9	0.9997	0.027	0.090
Genistein	0.1–10	*y* = 53.8*x* + 3.4	0.9998	0.037	0.12

**Table 3:** Recovery results of spiked puerarin, daidzin, daidzein and genistein in *Radix puerariae* (*n* = 6).

Compounds	Background Value (mg/kg)	Spiking Level (mg/kg)	Recovery (%)	RSD (%)
Puerarin	4366	2100	96.1	0.76
		4000	101.3	1.2
		6000	102.2	1.1
Daidzin	520	300	109.6	1.8
		500	105.3	2.0
		750	97.6	2.1
Daidzein	210	100	101.0	5.1
		200	94.9	4.6
		300	104.7	6.8
Genistein	25	14	102.2	7.7
		28	90.7	6.4
		42	90.5	6.1

**Table 4:** Contents of puerarin, daidzin, daidzein and genistein in *Radix puerariae* from different regions (*n* = 3).

Origins	Variety	Contents (mg/g)			
		Puerarin	Daidzin	Daidzein	Genistein
Linyi, Shandong	RPL	37.0 ± 0.5 *	9.15 ± 0.19	1.36 ± 0.17	0.15 ± 0.01
Shennongjia, Hubei	RPL	15.9 ± 0.7	2.21 ± 0.11	1.18 ± 0.01	0.010 ± 0.001
Shangluo, Shaanxi	RPL	80.4 ± 0.7	12.0 ± 0.1	1.02 ± 0.03	0.087 ± 0.004
Dabieshan, Anhui	RPL	78.6 ± 0.9	11.4 ± 0.2	0.99 ± 0.03	0.071 ± 0.003
Zhangjiajie, Hunan	RPL	78.3 ± 0.8	11.1 ± 0.1	1.12 ± 0.02	0.082 ± 0.001
Shangrao, Jiangxi	RPL	55.4 ± 1.9	9.72 ± 0.35	0.88 ± 0.04	0.082 ± 0.005
Chengdu, Sichuan	RPL	101.9 ± 0.7	14.9 ± 0.1	0.87 ± 0.03	0.072 ± 0.002
Bozhou, Anhui	RPT	4.14 ± 0.20	0.73 ± 0.02	0.14 ± 0.01	0.019 ± 0.003
Anqing, Anhui	RPT	4.37 ± 0.02	0.53 ± 0.01	0.21 ± 0.01	0.026 ± 0.004
Chuxiong, Yunnan	RPT	6.33 ± 0.16	1.02 ± 0.03	0.18 ± 0.01	0.021 ± 0.002
Wuzhou, Guangxi	RPT	4.21 ± 0.09	0.88 ± 0.01	0.22 ± 0.02	0.023 ± 0.003

* Average value ± $\frac{t\times s}{\sqrt{n}}$; *t* = 4.303 (*n* = 3, the confidence coefficient: 0.95), *s* denotes standard deviation.

## Data Availability

All related data and methods are presented in this paper. Additional inquiries should be addressed to the corresponding author.
